# 16S rRNA Gene Sequencing of Six Psyllid Species of the Family Carsidaridae Identified Various Bacteria Including *Symbiopectobacterium*

**DOI:** 10.1264/jsme2.ME23045

**Published:** 2023-08-22

**Authors:** Junnosuke Maruyama, Hiromitsu Inoue, Yuu Hirose, Atsushi Nakabachi

**Affiliations:** 1 Department of Applied Chemistry and Life Science, Toyohashi University of Technology, 1–1 Hibarigaoka, Tempaku, Toyohashi, Aichi 441–8580, Japan; 2 Institute for Plant Protection, National Agriculture and Food Research Organization, Higashihiroshima, Hiroshima 739–2494, Japan; 3 Research Institute for Technological Science and Innovation, Toyohashi University of Technology, 1–1 Hibarigaoka, Tempaku, Toyohashi, Aichi 441–8580, Japan

**Keywords:** *Symbiopectobacterium*, *Wolbachia* supergroup O, *Arsenophonus*, *Sodalis*, *Xanthomonas*

## Abstract

Psyllids (Hemiptera: Sternorrhyncha: Psylloidea) are plant sap-sucking insects that are closely associated with various microbes. To obtain a more detailed understanding of the ecological and evolutionary behaviors of microbes in Psylloidea, the bacterial populations of six psyllid species, belonging to the family Carsidaridae, were analyzed using high-throughput amplicon sequencing of the 16S rRNA gene. The majority of the secondary symbionts identified in the present study were gammaproteobacteria, particularly those of the order *Enterobacterales*, including *Arsenophonus* and *Sodalis*, which are lineages found in a wide variety of insect hosts. Additionally, *Symbiopectobacterium*, another *Enterobacterales* lineage, which has recently been recognized and increasingly shown to be vertically transmitted and mutualistic in various invertebrates, was identified for the first time in Psylloidea. This lineage is closely related to *Pectobacterium* spp., which are plant pathogens, but forms a distinct clade exhibiting no pathogenicity to plants. Non-*Enterobacterales* gammaproteobacteria found in the present study were *Acinetobacter*, *Pseudomonas* (both *Pseudomonadales*), *Delftia*, *Comamonas* (both *Burkholderiales*), and *Xanthomonas* (*Xanthomonadales*), a putative plant pathogen. Regarding alphaproteobacteria, three *Wolbachia* (*Rickettsiales*) lineages belonging to supergroup B, the major group in insect lineages, were detected in four psyllid species. In addition, a *Wolbachia* lineage of supergroup O, a minor group recently found for the first time in Psylloidea, was detected in one psyllid species. These results suggest the pervasive transfer of bacterial symbionts among animals and plants, providing deeper insights into the evolution of the interactions among these organisms.

Psyllids (Psylloidea) are plant sap-sucking hemipteran insects that encompass ~4,000 described species worldwide, constituting the suborder Sternorrhyncha along with whiteflies (Aleyrodoidea), aphids (Aphidoidea), phylloxera (Phylloxeroidea), and scale insects (Coccoidea) (Burckhardt *et al.*, 2021). Similar to other sternorrhynchan insects (International Aphid Genomics Consortium, 2010; Nakabachi and Miyagishima, 2010; Tamborindeguy *et al.*, 2010), psyllids feed exclusively on phloem sap using their needle-like mouthpart called the stylet (Hodkinson, 1974; Burckhardt *et al.*, 2021). Due to this feeding habit, some psyllid species transmit plant pathogens, including “*Candidatus* Liberibacter spp.” (*Alphaproteobacteria*: *Rhizobiales*) and “*Ca.* Phytoplasma spp.” (*Bacilli*: *Acholeplasmatales*), making them important agricultural pests (Jarausch and Jarausch, 2010; Grafton-Cardwell *et al.*, 2013; Mora *et al.*, 2021). Moreover, phloem sap is deficient in nutrients, including essential amino acids and some vitamins (Ziegler *et al.*, 1975; Sandström and Moran, 1999). Therefore, these deficiencies are compensated by transovarially transmitted bacterial mutualists harbored in a specialized organ called the bacteriome (Nakabachi *et al.*, 2010b; Sloan *et al.*, 2014). Although *in situ* hybridization has revealed the symbiont localization of only a few psyllid species (Fukatsu and Nikoh, 1998, 2000; Subandiyah *et al.*, 2000; Nakabachi *et al.*, 2013b; Dan *et al.*, 2017), the psyllid bacteriome is considered to typically harbor two distinct intracellular symbionts, based on microscopic observations with classical staining methods (Profft, 1937; Buchner, 1965), followed by bacterial gene amplicon ana­lyses with cloning (Spaulding and von Dohlen, 1998, 2001; Thao *et al.*, 2000a; Hansen *et al.*, 2007) and next-generation sequencing methods (Arp *et al.*, 2014; Hall *et al.*, 2016; Fromont *et al.*, 2017; Morrow *et al.*, 2017, 2020; Nakabachi *et al.*, 2020a, 2022a, 2022b), along with metagenomic ana­lyses (Sloan and Moran, 2012; Nakabachi *et al.*, 2013b, 2020b). The primary symbiont, the nutritional mutualist conserved among host species and, thus, considered to be essential in distinct insect clades, is “*Ca.* Carsonella ruddii” (*Gammaproteobacteria*: *Oceanospirillales*, hereafter *Carsonella*) in Psylloidea (Thao *et al.*, 2000b), which provides the host with essential amino acids (Nakabachi *et al.*, 2006; Sloan and Moran, 2012; Nakabachi *et al.*, 2013b, 2020b; Nakabachi and Moran, 2022). Molecular phylogenetic ana­lyses have repeatedly demonstrated cospeciation between host psyllids and *Carsonella*, resulting from the single acquisition of a *Carsonella* ancestor by a psyllid common ancestor and its subsequent stable vertical transmission (Thao *et al.*, 2000b; Spaulding and von Dohlen, 2001; Hall *et al.*, 2016; Nakabachi *et al.*, 2020a, 2022a, 2022b). Another bacterial lineage harbored in the bacteriome is categorized as a ‘secondary symbiont’, meaning an additional symbiont. Although secondary symbionts in diverse insect taxa form various host-symbiont relationships across the mutualism-parasitism continuum (Gherna *et al.*, 1991; Dale and Maudlin, 1999; Nakabachi *et al.*, 2003; Russell *et al.*, 2003; Thao and Baumann, 2004; Moran *et al.*, 2008; Werren *et al.*, 2008; Oliver *et al.*, 2010; Johnson, 2015), those in the psyllid bacteriome consistently appear to have obligate mutualistic relationships with the host psyllid (Sloan and Moran, 2012; Nakabachi *et al.*, 2013b, 2020b). Nevertheless, these secondary symbionts are phylogenetically diverse among host lineages, suggesting their repeated infection and replacement during the evolution of Psylloidea (Thao *et al.*, 2000a; Spaulding and von Dohlen, 2001; Sloan and Moran, 2012; Hall *et al.*, 2016; Morrow *et al.*, 2017; Nakabachi *et al.*, 2020a, 2022a, 2022b). They are mostly considered to have a nutritional basis (Spaulding and von Dohlen, 2001; Sloan and Moran, 2012; Morrow *et al.*, 2017), with the unique exception of‍ ‍“*Ca.* Profftella armatura” (*Gammaproteobacteria*: *Burkholderiales*) (Nakabachi *et al.*, 2013b, 2020a, 2020b; Dan *et al.*, 2017), the main role of which appears to protect the holobiont (host psyllid plus bacteriome-associated mutualists) from natural enemies, including predators, parasitoids, and pathogens (Nakabachi *et al.*, 2013b, 2020b; Nakabachi and Fujikami, 2019; Nakabachi and Okamura, 2019; Yamada *et al.*, 2019; Tanabe *et al.*, 2022). Besides these bacteriome-associated obligate mutualists, psyllids may harbor various secondary symbionts of a facultative nature, including *Wolbachia* (*Alphaproteobacteria*: *Rickettsiales*) and *Rickettsia* (*Alphaproteobacteria*: *Rickettsiales*), which cause systemic infection in host insects (Spaulding and von Dohlen, 2001; Sloan and Moran, 2012; Arp *et al.*, 2014; Chu *et al.*, 2016, 2019; Jain *et al.*, 2017; Kruse *et al.*, 2017; Morrow *et al.*, 2017; Nakabachi *et al.*, 2020a, 2022a, 2022b). Similar to other hemipteran insects (Nakabachi and Ishikawa, 1997, 1999, 2000, 2001; Nakabachi *et al.*, 2003, 2005, 2010a, 2014; Moran *et al.*, 2008; Kikuchi, 2009; Nikoh and Nakabachi, 2009; Gerardo *et al.*, 2010; Nikoh *et al.*, 2010; Ramsey *et al.*, 2010; Shigenobu *et al.*, 2010; Tamborindeguy *et al.*, 2010; Nakabachi, 2015; Uchi *et al.*, 2019), evidence is accumulating to show that interactions among symbiotic microbes, including those associated with the bacteriome, facultative symbionts, and plant pathogens, along with interactions between host psyllids and these bacterial populations (Nakabachi *et al.*, 2006, 2010b, 2013b; Sloan *et al.*, 2014; Dan *et al.*, 2017; Nakabachi and Fujikami, 2019), are important for psyllid biology and host plant pathology (Nakabachi *et al.*, 2013a, 2020a, 2022b; Chu *et al.*, 2016, 2019; Dan *et al.*, 2017; Jain *et al.*, 2017; Kruse *et al.*, 2017; Killiny, 2022; Tanabe *et al.*, 2022). Therefore, the identification of microbiomes in various psyllid lineages, which mirror the ecological and evolutionary behaviors of bacterial populations in Psylloidea, will help establish a basis for the better control of pest species.

According to a definition revised by Burckhardt *et al.*, psyllids are classified into seven extant families: Aphalaridae, Calophyidae, Carsidaridae, Liviidae, Mastigimatidae, Psyllidae, and Triozidae (Burckhardt *et al.*, 2021). Carsidaridae is a relatively small family consisting of three subfamilies, Carsidarinae, Homotominae, and Pachypsyllinae, which comprise 148 species of 23 genera (Ouvrard, 2023). This family encompasses agricultural pests, including *Mesohomotoma tessmanni* (Carsidarinae), *Allocarsidara malayensis* (Carsidarinae), and *Homotoma ficus* (Homotominae), which feed on cacao (Yana *et al.*, 2015), durian (Ketsa *et al.*, 2020), and fig (Tasi *et al.*, 2021) trees, respectively. A recent study performed a relatively comprehensive ana­lysis of 16S rRNA gene amplicons using 44 psyllid species of five families, including four Carsidaridae species: *H. ficus*, *Macrohomotoma gladiata* (Homotominae), *Mesohomotoma hibisci* (Carsidarinae), and *Protyora sterculiae* (Carsidarinae) (Kwak *et al.*, 2021). The ana­lysis revealed various bacteria, including two lineages of “*Ca*. Liberibacter” that are potential plant pathogens. One of them was a close relative of “*Ca*. Liberibacter asiaticus”, which is a notorious pathogen of the most devastating citrus disease, Huanglongbing or greening disease, and the other was “*Ca.* Liberibacter capsica”, a novel species that may potentially cause diseases in solanaceous crops. Although this study provided important insights into the evolution of microbiomes in Psylloidea, the ana­lysis was performed using (1) only one individual or a pool of two individuals for each psyllid species, making the ana­lysis less reliable; (2) primers unsuitable for detecting bacteria with AT-rich 16S rRNA genes, including *Carsonella*; and (3) the analytical method that clusters sequence reads with a similarity threshold of 97%, resulting in a lower resolution of the ana­lysis. Moreover, (4) none of the representative sequences were deposited in public databases, making phylogenetic ana­lyses difficult to reproduce.

In the present study, amplicon ana­lyses were performed based on Illumina sequencing of the V3 and V4 regions of 16S rRNA genes to assess the microbiomes of six Carsidaridae species collected in Japan (Table 1) using (1) ten (five adult males and five adult females) pooled individuals for each species, (2) primers optimized to detect 16S rRNA genes with a wider variety of G+C contents, and (3) the method to remove sequencing errors and resolve sequence variants (SVs) down to the level of single-nucleotide differences. Furthermore, (4) all of the main SVs (>1% of the total reads) have been deposited in public databases, providing the research community with a basis for the further investigation and reexamination of the analytical results.

## Materials and Methods

### Insect sampling and DNA extraction

Adults of six psyllid species belonging to the family Carsidaridae were collected from their host plants at various locations in Japan (Table 1). Insect samples were stored in acetone (*Carsidara limbata*, *Homotoma radiata*, and *Celtisaspis japonica*) or 99.5% ethanol (the other species) at –20°C until DNA extraction. DNA was extracted from the whole bodies of pooled individuals of five adult males and five adult females of each psyllid species using the DNeasy Blood & Tissue Kit (Qiagen). The quality of extracted DNA was assessed using a NanoDrop 2000c spectrophotometer (Thermo Fisher Scientific). Its quantity was assessed using a Qubit 2.0 fluorometer with the Qubit dsDNA HS Assay Kit (Thermo Fisher Scientific).

### Construction and sequencing of amplicon libraries

Bacterial populations in psyllids were analyzed in accordance with the instructions provided by Illumina (Illumina, 2013) with some modifications (Nakabachi *et al.*, 2020a, 2022a, 2022b). An amplicon polymerase chain reaction (PCR) was performed using DNA extracted from psyllids, KAPA HiFi HotStart ReadyMix (KAPA Biosystems), and the primer set 16S_341Fmod (5′-TCGTCGGCAGCGTCAGATGTGTATAAGAGACAG***YY***TA***M***GG***R***NGGCWGCAG-3′) and 16S_805R (5′-GTCTCGTGGGCTCGGAGATGTGTATAAGAGACAGGACTACHVGGGTATCTAATCC-3′), tar­geting the V3 and V4 regions of the 16S rRNA gene. Underlined areas indicate regions corresponding to target 16S rRNA gene sequences. Other nucleotides were the overhangs required for preparing and sequencing Illumina libraries. Although both primers were based on the instructions by Illumina (Illumina, 2013), 16S_341F was modified (bold italics), where the original CC, C, and G were replaced with the mixed bases YY (C or T), M (A or C), and R (A or G), respectively. This modification was based on an alignment of 16S rRNA genes encoded in divergent bacterial genomes with various G+C contents. Referenced sequences included those of 44 diverse lineages of *Carsonella* derived from 38 psyllid species from 23 genera, belonging to five families, which were available in the nucleotide database of the National Center for Biotechnology Information (NCBI) as of August 2014. The modification achieved a 100% match of the primers to most *Carsonella* sequences, retaining a single mismatch each in four lineages (Supplementary Fig. S1). In our reana­lysis in May 2023, we identified 11 more *Carsonella* sequences deposited in the NCBI nucleotide database, nine of which showed no mismatch to the primers. One sequence had a single mismatch and another showed two mismatches (single each for 16S_341Fmod and 16S_805R) (Supplementary Fig. S1). This modification increased sensitivity to detect symbionts with AT-rich genomes, including *Carsonella*, without reducing sensitivity to those with GC-rich genomes (Nakabachi *et al.*, 2020a, 2022a, 2022b). Dual indices and Illumina sequencing adapters were attached to the amplicons with index PCR using Nextera XT Index Kit v2 (Illumina). The libraries were combined with PhiX Control v3 (Illumina), and 250 bp of both ends were sequenced on the MiSeq platform (Illumina) with MiSeq Reagent Kit v2 (500 cycles; Illumina).

### Computational ana­lysis of bacterial populations

Output sequences were processed using the QIIME2 platform (version 2022.8) (Bolyen *et al.*, 2019). Primer sequences were removed from the demultiplexed sequence reads using the cutadapt plugin (Martin, 2011). The denoising and joining of paired-end reads and removal of low-quality or chimeric reads were subsequently performed using the DADA2 plugin (Callahan *et al.*, 2016). In this process, parameters were set to --p-trunc-len-f 230 and --p-trunc-len-r 225 to remove 3′-end nucleotides with quality scores below the Phred score 30, referring to the quality histograms drawn with fastQC (version 0.11.9) (Andrews, 2023) and multiQC (version1.12) (Ewels *et al.*, 2016). Dereplicated amplicon reads were classified, and taxonomic information was assigned using the q2-feature-classifier (Bokulich *et al.*, 2018), which was trained with the V3 and V4 regions of the 16S rRNA gene (Silva 138 SSURef NR99) (Glöckner *et al.*, 2017). The SVs obtained were manually checked by BLASTN searches against the NCBI non-redundant database (Camacho *et al.*, 2009). After SVs were aligned to related sequences using SINA (version 1.2.11) (Pruesse *et al.*, 2012), phylogenetic trees were inferred by the maximum likelihood (ML) method using RAxML (version 8.2.12) (Stamatakis, 2014). The GTR+Γ model was used with no partitioning of the data matrix, with 1,000 bootstrap iterations (options -f a -m GTRGAMMA -# 1000).

### Data availability

Nucleotide sequence data are available in the DDBJ/EMBL/GenBank databases under the accession numbers DRR420941–DRR420946 (raw fastq files) and TAAE01000001–TAAE01000035 (dereplicated SVs).

## Results

### Overview

**Fig. 1.**
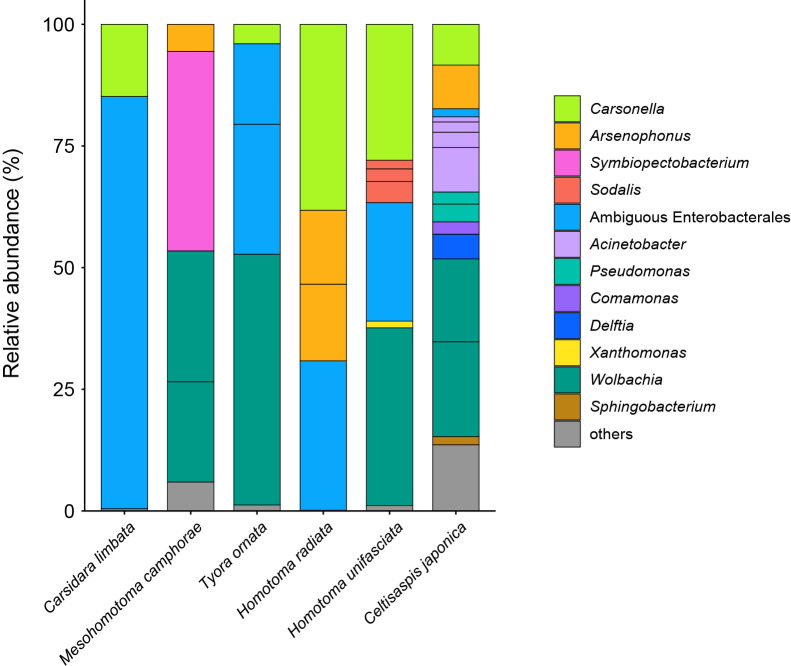
Composition of bacterial populations in psyllids of the family Carsidaridae. The relative abundance of Illumina reads belonging to assigned bacterial taxa are shown.

Raw libraries after demultiplexing yielded 48,874–83,223 pairs of forward and reverse reads for the six psyllid species. The denoising and joining of paired-end reads and removal of low-quality or chimeric reads resulted in 42,709–64,067 non-chimeric high-quality reads (Supplementary Table S1). The dereplication of these reads resulted in 342 independent SVs, 33 of which accounted for >1% of the total reads (Supplementary Table S2 and Fig. S2). The present study focused on the 33 main SVs unless otherwise noted because (1) filtering with a threshold of 1% was previously demonstrated to be among the most accurate and effective methods to eliminate potential contaminants (Karstens *et al.*, 2019) and (2) the targets of the present study were relatively abundant symbionts with close associations with the host psyllids. SVs with a relative abundance of <1% were collectively categorized as ‘others’ (Fig. 1) and accounted for 0.1 (*H. radiata*) - 13.6% (*C. japonica*) of the total reads in each psyllid species (Supplementary Table S2).

### Identification of *Carsonella*

**Fig. 2.**
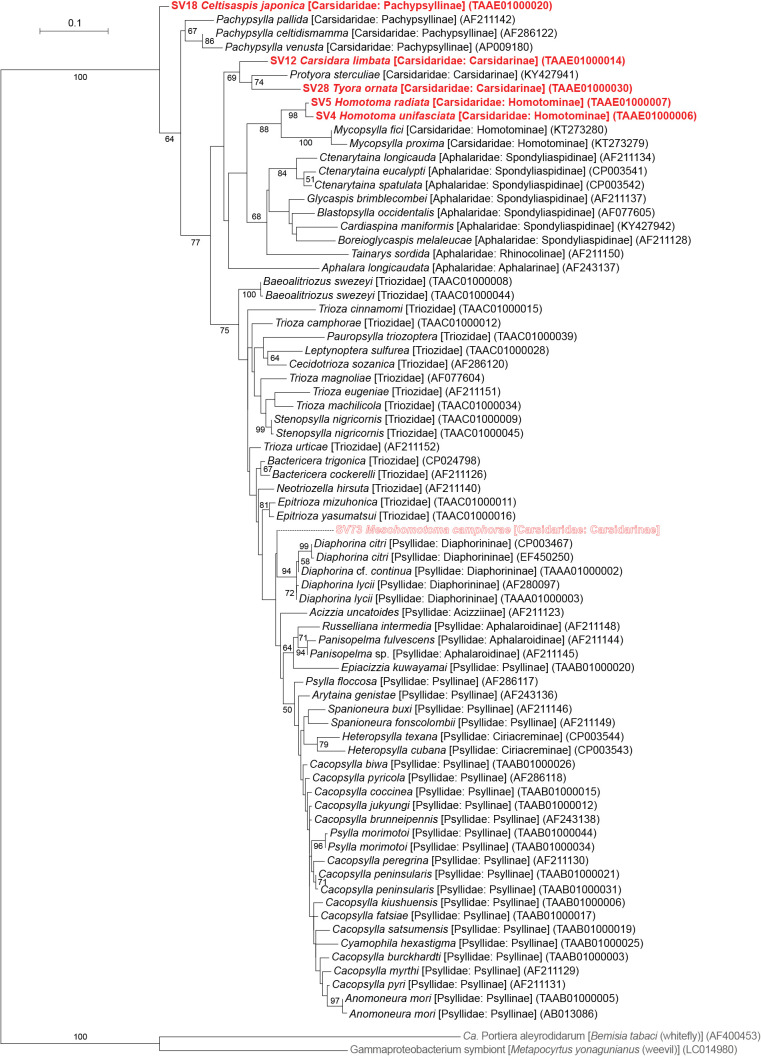
Maximum likelihood phylogram of *Carsonella*. A total of 427 unambiguously aligned nucleotide sites of 16S rRNA genes were subjected to the ana­lysis. On each branch, bootstrap support values of >50% are shown. Designations other than those for outgroups refer to psyllid hosts. Families and subfamilies (if applicable) of host psyllids are shown in brackets. Sequences from this study are shown in bold red. SV73 derived from *Mesohomotoma camphorae* is outlined. DDBJ/EMBL/GenBank accession numbers for sequences are provided in parentheses. The bar represents nucleotide substitutions per position. The outgroups were *Ca.* Portiera aleyrodidarum; the primary symbiont of the whitefly *Bemisia tabaci* (Hemiptera: Sternorrhyncha: Aleyrodoidea), and a gammaproteobacterium symbiont of the weevil *Metapocyrtus yonagunianus* (Coleoptera: Curculionidae).

The taxonomic classification by QIIME2 (Supplementary Table S2) followed by independent BLAST searches and phylogenetic ana­lyses showed that all psyllid species, except for *Mesohomotoma camphorae*, possess distinct lineages of *Carsonella* (Fig. 1). The ML tree placed these *Carsonella* sequences at positions that are largely consistent with the host psyllid phylogeny inferred by mitochondrial and nuclear gene ana­lyses with the aid of morphological ana­lyses (Percy *et al.*, 2018; Cho *et al.*, 2019; Burckhardt *et al.*, 2021) (Fig. 2). SV4 and SV5, derived from the congeneric species *Homotoma unifasciata* (27.9% of the reads) and *H. radiata* (38.2% of the reads; both Homotominae), respectively, formed a robustly supported clade (bootstrap: 98%). This clade further formed a well-supported clade (bootstrap: 88%) with other psyllid species belonging to the same subfamily Homotominae (Fig. 2). SV12 and SV28, derived from *C. limbata* (14.8% of the reads) and *Tyora ornata* (4.1% of the reads; both Carsidarinae), respectively, also formed a moderately supported clade (bootstrap: 69%) with a sequence from *P. sterculiae* (Carsidaridae: Carsidarinae, KY427941). SV18, which accounted for 8.4% of *C. japonica* (Pachypsyllinae) reads, was placed at the basal position in the *Carsonella* tree (Fig. 2). Although SV18 did not form a clade with sequences derived from other Pachypsyllinae species of the New World endemic genus *Pachypsylla*, the phylogenetic position was proximal to the moderately supported clade consisting of these sequences (bootstrap: 67%) (Fig. 2). These results were consistent with previous findings showing that host psyllids and *Carsonella* cospeciated due to the stable vertical transmission of *Carsonella* (Thao *et al.*, 2000b; Spaulding and von Dohlen, 2001; Hall *et al.*, 2016; Nakabachi *et al.*, 2020a, 2022a, 2022b).

In contrast, no *Carsonella*-like SVs with a relative abundance of ≥1% were detected in *M. camphorae* (Supplementary Table S2). This was unexpected because *Carsonella* was considered to be essential in Psylloidea, and the primer set (Supplementary Fig. S1) used in the present study was shown to be suitable for detecting *Carsonella* (Nakabachi *et al.*, 2020a, 2022a, 2022b). Therefore, the search was extended to include SVs with a relative abundance of <1% in *M. camphorae*. SV73, the most abundant SV in *M. camphorae* showing similarity to *Carsonella* sequences, accounted for only 0.16% of *M. camphorae* reads (Supplementary Table S2). The sequence was only 93.3% identical to the most similar sequences in the database, which were derived from *Carsonella* of *Diaphorina* cf. *continua* (AP023214, TAAA01000009) and *Diaphorina lycii* (TAAA01000012). These psyllid species belong to the family Psyllidae and are distantly related to *M. camphorae*. Moreover, the ML tree placed this SV at a position apart from *Carsonella* sequences found in other Carsidaridae species (Fig. 2). No sequence reads analyzed in the same MiSeq flow cell showed higher similarity to SV73 than the sequences found in *Diaphorina* spp. These results imply that SV73 was derived from a chimeric PCR artifact that QIIME2 failed to remove or a minor contaminant of an unknown source, not a true symbiont in *M. camphorae*. Similarly, all other *M. camphorae* SVs showing similarity to *Carsonella*, namely, SV125 (0.06% of the reads), SV213 (0.02%), SV219 (0.02%), and SV293 (0.01%), appeared to be derived from artifacts or contaminants. These types of minor SVs that showed similarity to *Carsonella* sequences, but appeared to correspond to PCR artifacts or contaminants of unknown sources were also detected in *C. limbata*, *T. ornata*, and *C. japonica* (Supplementary Table S2).

### *Enterobacterales* symbionts in Carsidaridae

**Fig. 3.**
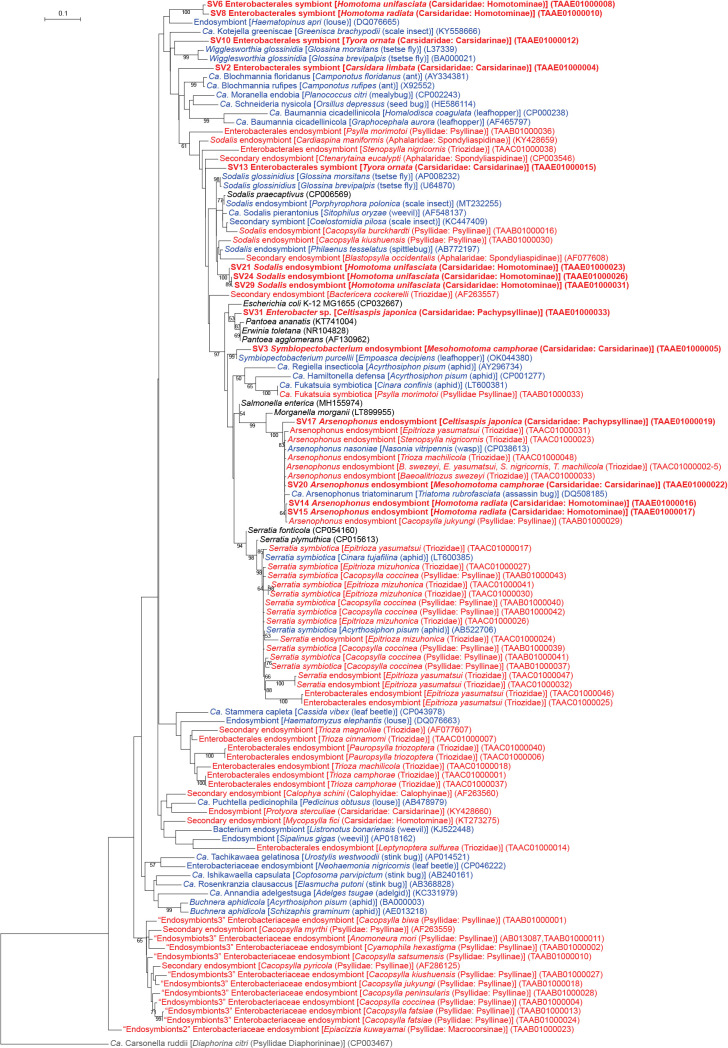
Maximum likelihood phylogram of *Enterobacterales*. A total of 427 unambiguously aligned nucleotide sites of 16S rRNA genes were subjected to the ana­lysis. On each branch, bootstrap support values of >50% are shown. The scale bar indicates substitutions per site. Regarding symbiotic bacteria, host organisms are shown in brackets. Symbionts of animals other than psyllids are shown in blue, while symbionts of psyllids are shown in red. Sequences from the present study are shown in bold. DDBJ/EMBL/GenBank accession numbers are provided in parentheses. *Carsonella* was used as an outgroup.

Of the 33 main SVs obtained in the present study, 28 corresponded to gammaproteobacteria, 14 of which belonged to the order *Enterobacterales* (Supplementary Table S2). *Enterobacterales* is a bacterial taxon that comprises numerous insect symbionts, including those associated with the bacteriome (Moran *et al.*, 2008; McCutcheon *et al.*, 2019). *Enterobacterales* bacteria identified in the present study included *Arsenophonus*, *Sodalis*, *Symbiopectobacterium*, and several lineages with ambiguous phylogenetic placements (Fig. 1 and 3, Supplementary Table S2).

### *Arsenophonus* symbionts

Four distinct SVs corresponding to *Arsenophonus* lineages were detected in three of the six Carsidaridae species: *H. radiata*, *C. japonica*, and *M. camphorae* (Fig. 1 and 3, Supplementary Table S2). SV14 (15.8% of *H. radiata* reads), SV15 (15.2% of *H. radiata* reads), SV17 (9.0% of *C. japonica* reads), and SV20 (5.5% of *M. camphorae* reads) were 97.2–100% identical to the sequences of *Arsenophonus nasoniae* (CP038613), the type species of *Arsenophonus* found in the parasitoid wasp *Nasonia vitripennis* (Hymenoptera: Pteromalidae) (Gherna *et al.*, 1991), and *Arsenophonus* symbionts detected in various insect lineages. Host insects included aphids (Russell *et al.*, 2003), whiteflies (Thao and Baumann, 2004), louse flies (Diptera: Hippoboscoidea) (Nováková *et al.*, 2009), and the psyllid species *Baeoalitriozus swezeyi*, *Epitrioza yasumatsui*, *Stenopsylla nigricornis*, *Trioza machilicola* (all Triozidae; TAAC01000002–5), *Cacopsylla jukyungi* (Psyllidae; TAAB01000029), *Cardiaspina tenuitela* (Aphalaridae; KY428657), and *Glycaspis brimblecombei* (Aphalaridae; EU043378) (Hansen *et al.*, 2007; Morrow *et al.*, 2017; Nakabachi *et al.*, 2022a, 2022b). These sequences formed a robustly supported clade (bootstrap: 100%) in the ML tree (Fig. 3). SV14 and SV15, both of which were derived from *H. radiata*, were 99.8% identical to each other. The similarities observed in both nucleotide sequences and read frequencies (see above) implied that these SVs corresponded to multiple copies of the 16S rRNA gene encoded in a single *Arsenophonus* genome. Although we cannot exclude the possibility that the nucleotide difference was caused by PCR/sequencing errors, the latter is less likely because the DADA2 plugin corrects sequencing errors during the denoising process (Callahan *et al.*, 2016; Prodan *et al.*, 2020).

### *Sodalis* symbionts

SV21, SV24, and SV29, which accounted for 4.3, 2.6, and 1.8%, respectively, of *H. unifasciata* reads (Supplementary Table S2), formed a clade with the *Sodalis* endosymbionts found in various insects, including the other psyllid species *Blastopsylla occidentalis* (Aphalaridae: Spondyliaspidinae; AF077608) (Spaulding and von Dohlen, 1998), *Cacopsylla burckhardti* (Psyllidae: Psyllinae; TAAB01000016), and *Cacopsylla kiushuensis* (Psyllidae: Psyllinae; TAAB01000030) (Fig. 3) (Nakabachi *et al.*, 2022b). These SVs were 96.5 (SV29)–97.0% (SV21) identical to the sequence of the type species *Sodalis glossinidius* (AP008232), a secondary symbiont of the tsetse fly *Glossina morsitans* (Diptera: Hippoboscoidea) (Dale and Maudlin, 1999). Although the clade was only poorly supported (bootstrap: <50%) (Fig. 3), these SVs were tentatively named “*Sodalis* endosymbionts” (Fig. 1 and 3) because their similarities to the type species were above the generally used arbitrary genus threshold of 94.5–95% (Yarza *et al.*, 2014; Barco *et al.*, 2020). SV21, SV24, and SV29 were 99.1–99.8% identical to one another.

### First report on a *Symbiopectobacterium* symbiont in Psylloidea

**Fig. 4.**
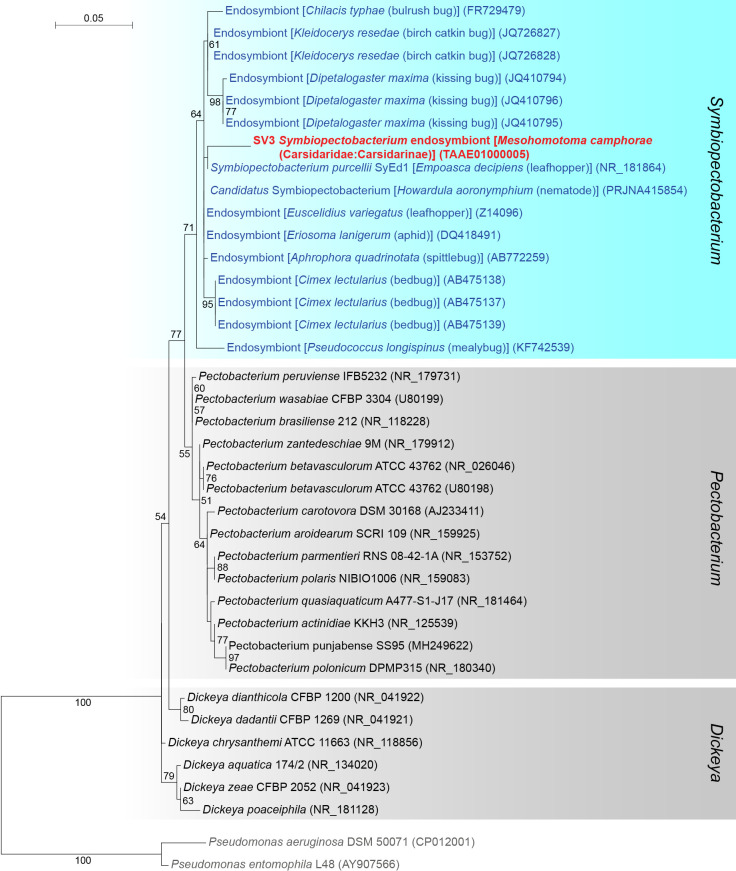
Maximum likelihood phylogram of *Symbiopectobacterium* and related genera. A total of 427 unambiguously aligned nucleotide sites of 16S rRNA genes were subjected to the ana­lysis. On each branch, bootstrap support values of >50% are shown. The scale bar indicates substitutions per site. Regarding symbiotic bacteria, host organisms are shown in brackets. Symbionts of animals other than psyllids are shown in blue, while symbionts of psyllids are shown in red. The sequence from the present study is shown in bold. DDBJ/EMBL/GenBank accession numbers are provided in parentheses. *Pseudomonas aeruginosa* and *Pseudomonas entomophila* were used as an outgroup.

SV3, which accounted for as much as 41.0% of *M. camphorae* reads (Supplementary Table S2), was 97.4% identical to the sequence of *Symbiopectobacterium purcellii*, an endosymbiont found in the leafhopper *Empoasca decipiens* (CP081864) (Nadal-Jimenez *et al.*, 2022b). These sequences formed a robustly supported clade (bootstrap: 99%) in the ML tree of the order *Enterobacterales* (Fig. 3). Since the genus *Symbiopectobacterium* is closely related to the genus *Pectobacterium*, which includes important plant pathogens (Rossmann *et al.*, 2018; Oulghazi *et al.*, 2021), we performed a more comprehensive ana­lysis that focused on *Symbiopectobacterium* and related bacterial genera. The results obtained placed SV3 in a well-supported clade (bootstrap: 71%) of vertically transmitted *Symbiopectobacterium* symbionts recently recognized and identified in various invertebrate lineages (Fig. 4) (Hosokawa *et al.*, 2010; Kuechler *et al.*, 2011, 2012; da Mota *et al.*, 2012; Koga *et al.*, 2013; Lopez-Madrigal *et al.*, 2014; Husnik and McCutcheon, 2016; Martinson *et al.*, 2020; Nadal-Jimenez *et al.*, 2022b). This clade is distinct from that of *Pectobacterium* spp., which are plant pathogens that cause soft rot disease in various economically important crops (Rossmann *et al.*, 2018; Oulghazi *et al.*, 2021) (Fig. 4). Bacteria belonging to this newly emerging *Symbiopectobacterium* clade have mainly been discovered in hemipteran insects, including plant-sap feeders and vertebrate-blood feeders. However, to the best of our knowledge, this is the first study to report *Symbiopectobacterium* in Psylloidea.

### Other *Enterobacterales* symbionts

SV2 (84.7% of *C. limbata* reads), SV6 (24.4% of *H. unifasciata* reads), SV8 (30.7% of *H. radiata* reads), SV10 (26.2% of *T. ornata* reads), and SV13 (16.6% of *T. ornata* reads) were placed at ambiguous positions in the ML tree (Fig. 1 and 3, Supplementary Table S2). Since their branching patterns were mostly poorly supported (bootstrap: <50%) and their sequence identities with those of bacteria with a genus name were low (<94.5%), they were collectively referred to as “*Enterobacterales* symbionts” (Fig. 1 and 3). Among them, SV6 and SV8, which were derived from congeneric psyllid species, formed a robustly supported clade (bootstrap: 100%) in the ML tree (Fig. 3), suggesting that the corresponding symbionts are sister lineages that share a common ancestor in the common ancestral host. This phylogenetic relationship implies the important and conserved roles of these symbionts in host psyllids. SV31, which accounted for 1.7% of *C. japonica* reads, was 100% identical to the sequence of *Enterobacter* spp. (Fig. 1 and 3, Supplementary Table S2). This low relative abundance and 100% identity with free-living bacteria implied that the corresponding bacterium was a transient associate, not a stable symbiont.

### Bacteria of *Pseudomonadales*, *Burkholderiales*, and *Xanthomonadales*

**Fig. 5.**
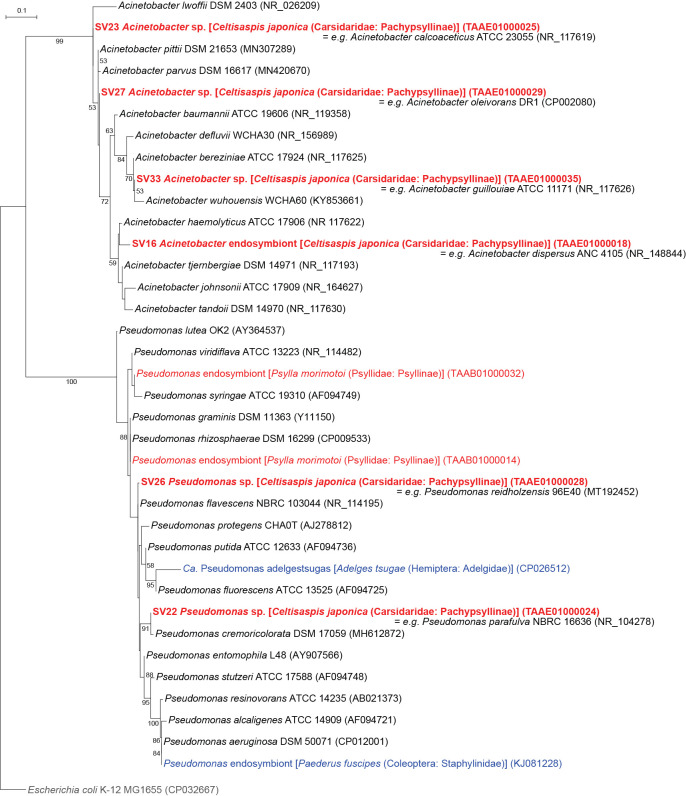
Maximum likelihood phylogram of *Pseudomonadales*. A total of 427 unambiguously aligned nucleotide sites of 16S rRNA genes were subjected to the ana­lysis. On each branch, bootstrap support values of >50% are shown. The scale bar indicates substitutions per site. Regarding symbiotic bacteria, host organisms are shown in brackets. Symbionts of animals other than psyllids are shown in blue, while symbionts of psyllids are shown in red. The sequence from the present study is shown in bold. DDBJ/EMBL/GenBank accession numbers are provided in parentheses. *Escherichia coli* was used as an outgroup.

Six SVs detected in *C. japonica* were placed in the clade of the order *Pseudomonadales* (Fig. 1 and 5, Supplementary Table S2). SV16, SV23, SV27, and SV33, which accounted for 9.2, 3.2, 2.1, and 1.1%, respectively, of *C. japonica* reads, were resolved as distinct lineages in the robustly supported clade (bootstrap: 99%) of the genus *Acinetobacter* (Fig. 5). SV22 and SV26, which accounted for 3.7 and 2.5%, respectively, of *C. japonica* reads, were shown as distinct lineages in the robustly supported clade (bootstrap: 100%) of the genus *Pseudomonas* (Fig. 5).

**Fig. 6.**
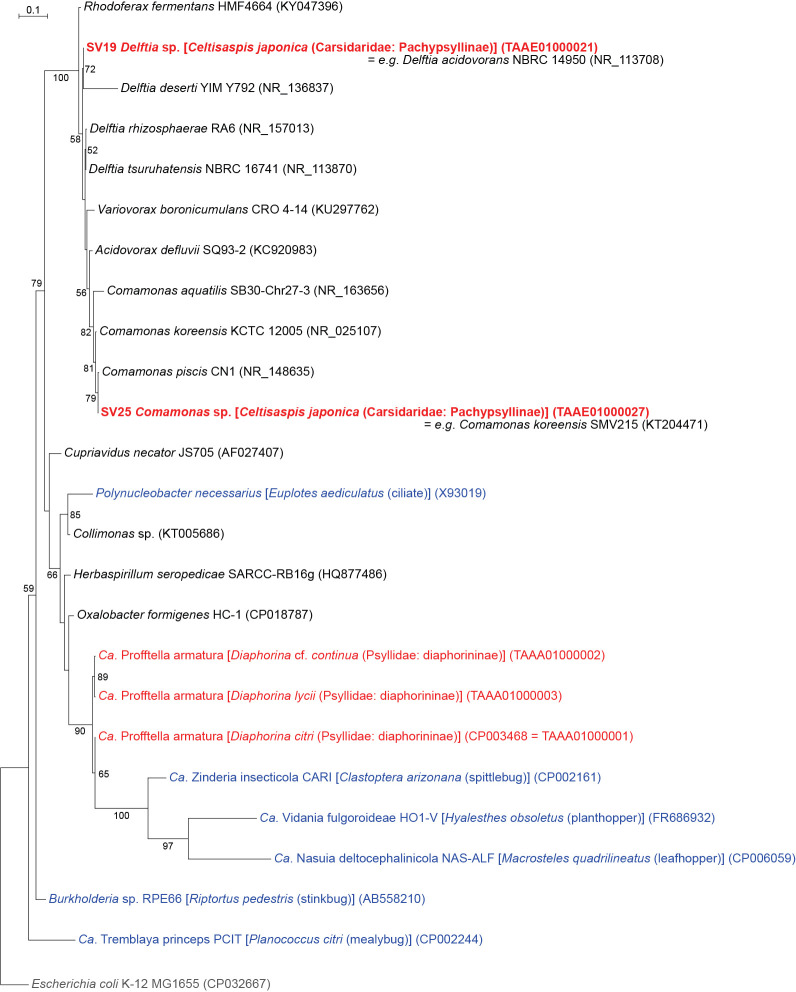
Maximum likelihood phylogram of *Burkholderiales*. A total of 427 unambiguously aligned nucleotide sites of 16S rRNA genes were subjected to the ana­lysis. On each branch, bootstrap support values of >50% are shown. The scale bar indicates substitutions per site. Regarding symbiotic bacteria, host organisms are shown in brackets. Symbionts of animals other than psyllids are shown in blue, while symbionts of psyllids are shown in red. The sequence from the present study is shown in bold. DDBJ/EMBL/GenBank accession numbers are provided in parentheses. *Escherichia coli* was used as an outgroup.

SV19 and SV25, which accounted for 5.1 and 2.5%, respectively, of *C. japonica* reads, were inferred as distinct lineages in the robustly supported clade (bootstrap: 100%) within the order *Burkholderiales* (Fig. 1 and 6, Supplementary Table S2). Although *Profftella*, a unique organelle-like defensive symbiont (Nakabachi *et al.*, 2013b), also belongs to this order, the ML tree showed that these SVs were distantly related to *Profftella*. The SVs placed in *Pseudomonadales* or *Burkholderiales* were 100% identical to the sequences of free-living species, and their abundance was relatively low. Therefore, these SVs may be derived from transient associates, not stable symbionts of *C. japonica*.

SV32, which accounted for 1.4% of *H. unifasciata* reads, was 100% identical to the sequence of *Xanthomonas* spp. (*Xanthomonadales*), including *X. campestris* (type species), *X. oryzae*, *X. citri*, *X. arboricola*, *X. hortorum*, and *X. vasicola*, which are pathogens of important agricultural crops (Timilsina *et al.*, 2020) (Fig. 1 and Supplementary Table S2).

### *Wolbachia* of supergroups B and O

**Fig. 7.**
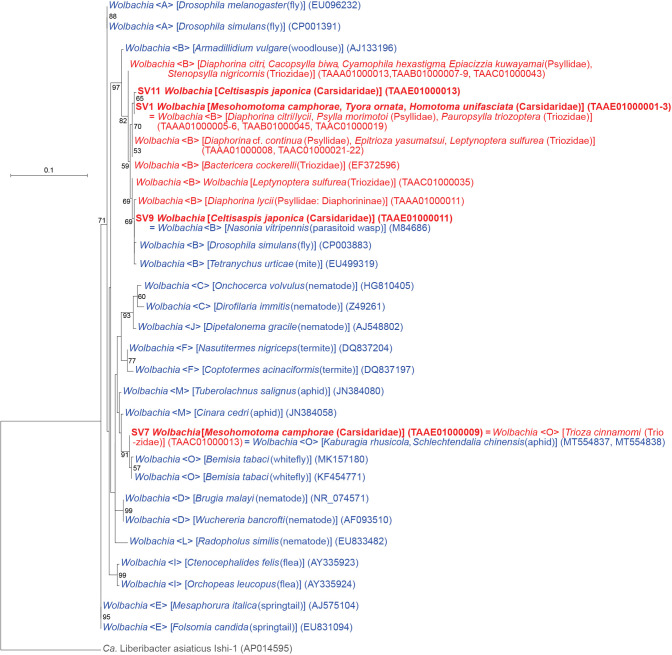
Maximum likelihood phylogram of *Wolbachia*. A total of 402 unambiguously aligned nucleotide sites of 16S rRNA genes were subjected to the ana­lysis. On each branch, bootstrap support values of >50% are shown. Host organisms are shown in brackets. Symbionts of animals other than psyllids are shown in blue, while symbionts of psyllids are shown in red. The sequence from this study is shown in bold. DDBJ/EMBL/GenBank accession numbers for sequences are provided in parentheses. Supergroups of *Wolbachia* are shown in angle brackets. The scale bar represents nucleotide substitutions per position. *Liberibacter* was used as an outgroup.

The ana­lysis identified four SVs corresponding to distinct lineages of *Wolbachia* (*Alphaproteobacteria*: *Rickettsiales*) (Fig. 1 and 7, Supplementary Table S2), which are rickettsial bacteria found in various arthropods and nematodes (Werren *et al.*, 2008). *Wolbachia* lineages are currently classified into supergroups A–Q (Lindsey *et al.*, 2016). Supergroups A and B are the most widespread supergroups in arthropods, and most *Wolbachia* strains previously found in psyllids belonged to supergroup B (Spaulding and von Dohlen, 2001; Sloan and Moran, 2012; Arp *et al.*, 2014; Jain *et al.*, 2017; Morrow *et al.*, 2017; Chu *et al.*, 2019; Nakabachi *et al.*, 2020a, 2022a, 2022b). In contrast, SV7, which accounted for 26.9% of *M. camphorae* reads (Supplementary Table S2), was 100% identical to the sequence of *Wolbachia* belonging to supergroup O. This sequence was recently detected in another psyllid species *Trioza cinnamomi* (Triozidae), which was the first report of this supergroup in Psylloidea (Nakabachi *et al.*, 2022a). Moreover, the sequence was identical to that of *Wolbachia* detected in two aphid species, *Kaburagia rhusicola* (MT554837) and *Schlechtendalia chinensis* (MT554838) (Ren *et al.*, 2020). The ML ana­lysis placed the sequence within a robustly supported clade (bootstrap: 91%) of *Wolbachia* supergroup O (Fig. 7). All other *Wolbachia* strains found in the present study (SV1, SV9, and SV11) belonged to supergroup B (Fig. 7).

## Discussion

The present study identified various bacterial populations in six psyllid species of the family Carsidaridae collected in Japan. Although distinct lineages of *Carsonella* were identified in five psyllid species, essentially no sequence for *Carsonella* was detected in *M. camphorae* (Fig. 1 and 2, Supplementary Table S2). The universal primers suggested by Illumina (Illumina, 2013) tend to fail to detect AT-rich 16S rRNA genes of *Carsonella* (Fromont *et al.*, 2017; Morrow *et al.*, 2017, 2020; Kwak *et al.*, 2021). However, the primer set used in the present study was modified to detect sequences with a wider variety of G+C contents (Supplementary Fig. S1) and successfully identified *Carsonella* in diverse psyllid lineages (Nakabachi *et al.*, 2020a, 2022a, 2022b). Therefore, the present results may imply the absence of *Carsonella* at least in the *M. camphorae* individuals analyzed in the present study. However, the failure of PCR detection may also be attributed to other reasons, including exceptionally diverged nucleotide sequences at primer annealing sites. Further studies, including comprehensive histological ana­lyses of a large set of specimens from several sampling points, are required to establish whether *M. camphorae* truly lacks *Carsonella*.

As shown in other psyllid lineages (Thao *et al.*, 2000a; Spaulding and von Dohlen, 2001; Sloan and Moran, 2012; Hall *et al.*, 2016; Morrow *et al.*, 2017; Nakabachi *et al.*, 2020a, 2022a, 2022b; Kwak *et al.*, 2021), the majority of secondary symbionts identified in the present study belong to *Gammaproteobacteria*, particularly the order *Enterobacterales* (Fig. 1 and 3, Supplementary Table S2). These include *Arsenophonus*, *Sodalis*, and *Symbiopectobacterium*, as well as several lineages with ambiguous phylogenetic placements. Although *Arsenophonus* and *Sodalis* are among the symbionts most frequently observed in arthropod hosts including psyllid lineages (Thao *et al.*, 2000a; Spaulding and von Dohlen, 2001; Hansen *et al.*, 2007; Moran *et al.*, 2008; Sloan and Moran, 2012; Arp *et al.*, 2014; Hall *et al.*, 2016; Morrow *et al.*, 2017; Nakabachi *et al.*, 2022a, 2022b), *Symbiopectobacterium* is the lineage identified for the first time in Psylloidea. *Arsenophonus* have been found in diverse insect groups, including wasps (Gherna *et al.*, 1991; Nadal-Jimenez *et al.*, 2023), bees (Hymenoptera: Apoidea) (Nadal-Jimenez *et al.*, 2022a), aphids (Russell *et al.*, 2003; Wulff and White, 2015; Tian *et al.*, 2019; Zhang *et al.*, 2021; Yorimoto *et al.*, 2022), psyllids (Spaulding and von Dohlen, 1998, 2001; Subandiyah *et al.*, 2000; Thao *et al.*, 2000a; Hansen *et al.*, 2007; Hall *et al.*, 2016; Morrow *et al.*, 2017; Nakabachi *et al.*, 2022a, 2022b), whiteflies (Thao and Baumann, 2004; Chiel *et al.*, 2007; El Hamss *et al.*, 2021), triatomine bugs (Hemiptera: Heteroptera) (Hypsa and Dale, 1997), lice (Psocodea: Anoplura) (Sasaki-Fukatsu *et al.*, 2006; Allen *et al.*, 2007; Kirkness *et al.*, 2010), louse flies (Dale *et al.*, 2006; Nováková *et al.*, 2009, 2016), and ticks (Arachnida: Ixodida) (Grindle *et al.*, 2003). The types of symbiotic relationships with hosts exhibit a wide diversity from parasitism (*e.g.* killing male progeny to drive the spread of maternally inherited symbionts in the host population [Gherna *et al.*, 1991]) through facultative mutualism (*e.g.* increasing the host population growth rate possibly through nutrient supplementation [Wulff and White, 2015; Tian *et al.*, 2019]) to bacteriome-associated obligate mutualism in order to provide essential nutrients to the host (*e.g.* essential amino acids and B vitamins for sap feeders and blood feeders, respectively) (Dale *et al.*, 2006; Sasaki-Fukatsu *et al.*, 2006; Allen *et al.*, 2007; Nováková *et al.*, 2009, 2016; Kirkness *et al.*, 2010; Yorimoto *et al.*, 2022). Phylogenetic ana­lyses by Nováková *et al.* revealed two contrasting evolutionary patterns in *Arsenophonus* lineages: random associations with distantly related hosts in diverse insect taxa and host-symbiont co-cladogenesis in the lineages of lice and louse flies (Nováková *et al.*, 2009). These findings imply at least two transitions from facultative symbionts capable of infecting new host lineages to obligate‍ ‍mutualists that have lost this ability. *Sodalis* symbionts have also been found in various insect taxa, including tsetse flies (Dale and Maudlin, 1999), weevils (Coleoptera: Curculionoidea) (Toju *et al.*, 2013; Oakeson *et al.*, 2014), psyllids (Arp *et al.*, 2014; Hall *et al.*, 2016; Morrow *et al.*,‍ ‍2017; Nakabachi *et al.*, 2022b), scale insects (Dhami *et‍ ‍al.*, 2013; Husnik and McCutcheon, 2016), spittlebugs (Hemiptera: Auchenorrhyncha: Cercopoidea) (Koga *et al.*, 2013; Koga and Moran, 2014), stinkbugs (Hemiptera: Heteroptera) (Kaiwa *et al.*, 2010), lice (Fukatsu *et al.*, 2007), and louse flies (Nováková and Hypša, 2007). Although the symbiotic types of *Sodalis* vary, their main role appears to be the provision of nutrients in many insect hosts (McCutcheon *et al.*, 2019). *Sodalis* symbionts were hypo­thesized to have replaced more ancient antecedent symbionts in spittlebugs (Koga *et al.*, 2013; Koga and Moran, 2014) and weevils (Toju *et al.*, 2013; Oakeson *et al.*, 2014). Physiological and genomic ana­lyses have suggested they provide the hosts with nutrients formerly supplied by the antecedents (Koga and Moran, 2014; Oakeson *et al.*, 2014).‍ ‍Since similar symbiont replacements by *Sodalis* have‍ ‍been suggested in psyllid lineages (Nakabachi *et al.*, 2022b), their functional roles in Psylloidea are of interest. The clade of *Symbiopectobacterium* was relatively recently recognized in comparison to *Arsenophonus* and *Sodalis* (Martinson *et al.*, 2020). Bacteria belonging to this clade have mainly been found in hemipteran insects. These encompass not only plant-feeding taxa, including aphids, mealybugs (Sternorrhyncha: Coccoidea) (Lopez-Madrigal *et al.*, 2014; Husnik and McCutcheon, 2016), leafhoppers (Auchenorrhyncha: Membracoidea) (Nadal-Jimenez *et al.*, 2022b), spittlebugs (Koga *et al.*, 2013), and seed bugs (Heteroptera) (Kuechler *et al.*, 2011, 2012), but also blood-feeding taxa, including kissing bugs (da Mota *et al.*, 2012) and bedbugs (both Heteroptera) (Hosokawa *et al.*, 2010). A‍ ‍recent ana­lysis discovered *Symbiopectobacterium* in the‍ ‍nematode *Howardula aoronymphium* (Secernentea: Tylenchida) that parasitizes *Drosophila* flies (Diptera: Drosophilidae) (Martinson *et al.*, 2020). *Symbiopectobacterium* symbionts are vertically transmitted through host generations, and lineages in mealybugs, seed bugs, and nematodes were demonstrated to be obligate mutualists (Kuechler *et al.*, 2011, 2012; Husnik and McCutcheon, 2016; Martinson *et al.*, 2020). All of the clades of *Arsenophonus* (Zreik *et al.*, 1998; Bressan, 2014), *Sodalis* (Chari *et al.*, 2015; Tláskal *et al.*, 2021), and *Symbiopectobacterium* (Leite *et al.*, 2023) include lineages that have close associations with plants, implying their evolutionary history of horizontal transfer among insects, at‍ ‍least partly through plants. Since the physiological and‍ ‍ecological roles of *Arsenophonus*, *Sodalis*, and *Symbiopectobacterium* symbionts identified in Psylloidea remain unknown, future studies need to focus on clarifying their localization within the insect body (*e.g.* whether they are harbored in the bacteriome) and their genomic structures to obtain an understanding of their metabolic potential.

Regarding *Wolbachia* (*Rickettsiales*), the present study identified three lineages belonging to supergroup B, the major group in insect lineages, in four psyllid species. Moreover, supergroup O, another *Wolbachia* lineage, which is a relatively minor taxon and was recently found for the first time in Psylloidea (Nakabachi *et al.*, 2022a), was identified in *M. camphorae*. This implies that supergroup O is widespread in Psylloidea, although it has only recently been recognized. *Wolbachia* is among the most widely distributed symbionts worldwide, estimated to infect 40% of terrestrial arthropods (Werren *et al.*, 2008; Brinker *et al.*, 2019). Although limited lineages are recognized to be obligate mutualists (Hosokawa *et al.*, 2010), they are mostly parasites in insects, in which they manipulate host reproduction to drive their dissemination (Werren *et al.*, 2008; Brinker *et al.*, 2019). They induce resistance to various microbes, including viruses and protozoans, in some insect taxa (Brinker *et al.*, 2019; Wang *et al.*, 2021). Moreover, a genome editing technique using the CRISPR-Cas9 system to transform *Wolbachia* has recently been developed (Pelz-Stelinski, 13 May 2021, United States Patent and Trademark Office). Therefore, they are regarded as promising agents in the control of pest insects and the microbes that they transmit (Brinker *et al.*, 2019; Pelz-Stelinski, 13 May 2021, United States Patent and Trademark Office; Wang *et al.*, 2021). A high incidence of *Wolbachia* in pest psyllids worldwide (Spaulding and von Dohlen, 2001; Sloan and Moran, 2012; Arp *et al.*, 2014; Chu *et al.*, 2016, 2019; Morrow *et al.*, 2017; Nakabachi *et al.*, 2020a, 2022b) and the interactions observed between *Wolbachia* and other symbionts (Chu *et al.*, 2016, 2019; Jain *et al.*, 2017; Kruse *et al.*, 2017; Killiny, 2022) have led researchers to anticipate using *Wolbachia* to manipulate pest psyllids (*e.g.* providing them with resistance to plant pathogens) and/or plant pathogens (*e.g.* preventing them from infecting plants) (Chu *et al.*, 2016, 2019; Kruse *et al.*, 2017; Pelz-Stelinski, 13 May 2021, United States Patent and Trademark Office). The present study suggested the pervasive horizontal transmission of various *Wolbachia* strains among various insects, including psyllids (Fig. 7). This implies that *Wolbachia* may be artificially infected into psyllids, forming the basis for the use of this bacterial group for application purposes.

Furthermore, the present study detected potential plant pathogens, including *Xanthomonas* sp., in *H. unifasciata*. Since *H. unifasciata* feeds on *Ficus* spp. (Moraceae), further studies are warranted to establish whether the host plants are infected with *Xanthomonas* and if the infection causes disease symptoms.

## Conclusions

The present study identified various bacterial symbionts in six psyllid species of the family Carsidaridae. The majority of the secondary symbionts were gammaproteobacteria, particularly those of the order *Enterobacterales*, including *Arsenophonus* and *Sodalis*. In addition, *Symbiopectobacterium*, another lineage belonging to *Enterobacterales*, was detected for the first time in Psylloidea. Regarding non-*Enterobacterales* gammaproteobacteria, *Acinetobacter*, *Pseudomonas* (both‍ ‍*Pseudomonadales*), *Delftia*, *Comamonas* (both *Burkholderiales*), and *Xanthomonas* (*Xanthomonadales*), a putative plant pathogen, were identified. Regarding alphaproteobacteria, three *Wolbachia* (*Rickettsiales*) lineages belonging to supergroup B, the major group in insect lineages, were detected in four psyllid species. Moreover, a *Wolbachia* lineage of supergroup O, a minor group that was recently found for the first time in Psylloidea, was detected in *M. camphorae*, suggesting that this supergroup is widespread in Psylloidea. These results provide deeper insights into the interactions among insects, bacteria, and plants, which will help establish a basis for the better control of pest species.

## Citation

Maruyama, J., Inoue, H., Hirose, Y., and Nakabachi, A. (2023) 16S rRNA Gene Sequencing of Six Psyllid Species of the Family Carsidaridae Identified Various Bacteria Including *Symbiopectobacterium*. *Microbes Environ ***38**: ME23045.

https://doi.org/10.1264/jsme2.ME23045

## Supplementary Material

Supplementary Material 1

Supplementary Material 2

Supplementary Material 3

## Figures and Tables

**Table 1.** Psyllid species used in the present study

Species	Subfamily	Sampling site	Collection date	Host plant
*Carsidara limbata*	Carsidarinae	Hakozaki, Fukuoka city, Fukuoka Pref., Kyushu, Japan (33.6262 N 130.4248 E)	November 15, 2015	*Firmiana simplex* (Malvaceae)
*Mesohomotoma camphorae*	Carsidarinae	Gusuku, Sumiyô, Amami-oshima Is., Kagoshima Pref., Ryukyus, Japan (28.2956 N 129.4572 E)	May 23, 2009	*Hibiscus hamabo* (Malvaceae)
*Tyora ornata*	Carsidarinae	Komi, Iriomote Is., Okinawa Pref., Ryukyus, Japan (24.3129 N 123.9053 E)	May 5, 2000	*Heritiera littoralis* (Malvaceae)
*Homotoma radiata*	Homotominae	Oniike, Itsuwa-machi, Amakusa City, Kumamoto Pref., Amakusa-shimoshima Is., Kyushu, Japan (32.5470 N 130.1865 E)	April 9, 2015	*Ficus subpisocarpa* (Moraceae)
*Homotoma unifasciata*	Homotominae	Fukuregi, Amakusa city, Kumamoto Pref., Amakusa-shimoshima Is., Kyushu, Japan (32.4029 N 130.0800 E)	May 22, 2013	*Ficus erecta* (Moraceae)
*Celtisaspis japonica*	Pachypsyllinae	Edosaki, Inasiki city, Ibaraki Pref., Honshu, Japan (35.9518 N 140.3216 E)	May 27, 2016	*Celtis sinensis* (Cannabaceae)
