# Supplementary material for: 16S rRNA Gene Sequencing of Six Psyllid Species of the Family Carsidaridae Identified Various Bacteria Including Symbiopectobacterium
Source: Microbes Environ. 2023 Aug 22;38(3):ME23045. doi: 10.1264/jsme2.ME23045 (PMC10522848; doi:10.1264/jsme2.ME23045)
Supplement: Supplementary file 1 — Supplementary Material 1 [file 38_23045_s1.pdf]

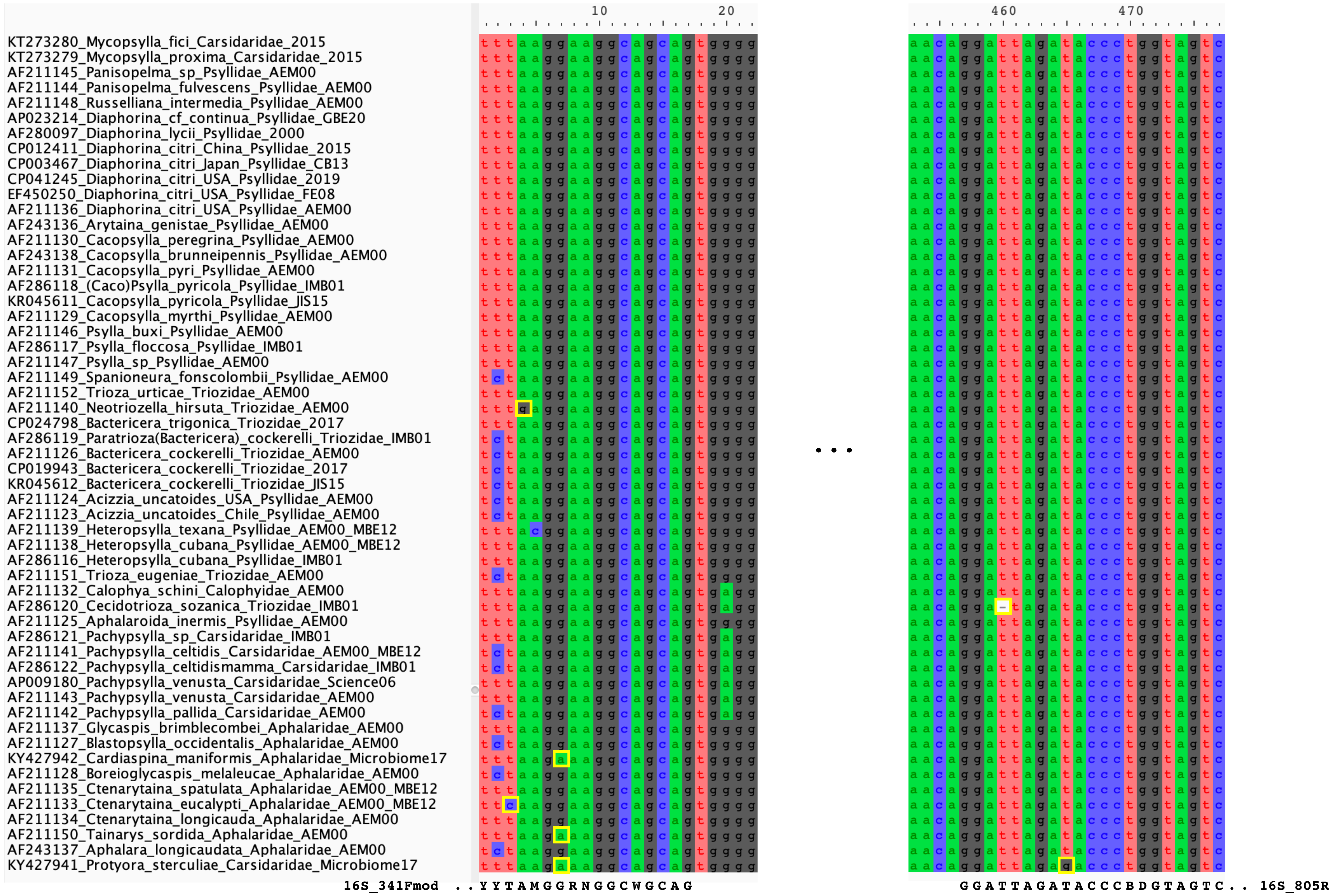

Supplementary Fig. S1. Alignment of 16S rRNA genes of *Carsonella* showing a limited number of mismatches to the primer set used for the present study. 16S rRNA gene sequences derived from 55 lineages of *Carsonella*, for which nucleotide sequences of primer target sites were available, were obtained from the NCBI database, aligned with MAFFT, and are shown with AliView. Primer sequences corresponding to target sites are shown at the bottom. Yellow squares denote mismatches to the primers.

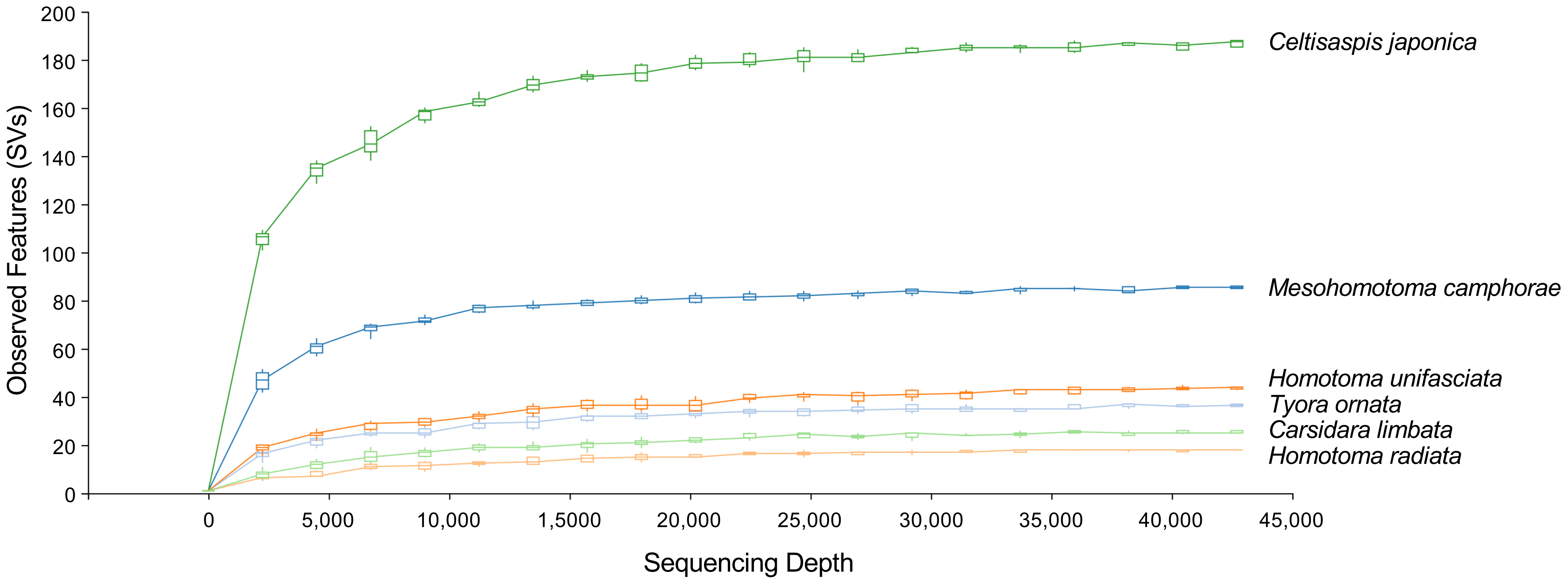

Supplementary Fig. S2. Rarefaction curves of specimens analyzed in the present study.
